# A two-step deconvolution-analysis-informed population pharmacodynamic modeling approach for drugs targeting pulsatile endogenous compounds

**DOI:** 10.1007/s10928-017-9526-0

**Published:** 2017-05-11

**Authors:** Michiel J. van Esdonk, Jacobus Burggraaf, Piet H. van der Graaf, Jasper Stevens

**Affiliations:** 1 Division of Pharmacology, Cluster Systems Pharmacology, Leiden Academic Centre for Drug Research, Leiden University, Leiden, The Netherlands; 2 Centre for Human Drug Research, Leiden, The Netherlands; 3 Certara QSP, Canterbury, UK; 4 Department of Clinical Pharmacy and Pharmacology, University Medical Center Groningen, Groningen, The Netherlands

**Keywords:** Population PK/PD modeling, Growth hormone, Deconvolution analysis, Pulsatile secretion

## Abstract

Pharmacodynamic modeling of pulsatile endogenous compounds (e.g. growth hormone [GH]) is currently limited to the identification of a low number of pulses. Commonly used pharmacodynamic models are not able to capture the complexity of pulsatile secretion and therefore non-compartmental analyses are performed to extract summary statistics (mean, AUC, C_max_). The aim of this study was to develop a new quantification method that deals with highly variable pulsatile data by using a deconvolution-analysis-informed population pharmacodynamic modeling approach. Pulse frequency and pulse times were obtained by deconvolution analysis of 24 h GH profiles. The estimated pulse times then informed a non-linear mixed effects population pharmacodynamic model in NONMEM V7.3. The population parameter estimates were used to perform simulations that show agonistic and antagonistic drug effects on the secretion of GH. Additionally, a clinical trial simulation shows the application of this method in the quantification of a hypothetical drug effect that inhibits GH secretion. The GH profiles were modeled using a turnover compartment in which the baseline secretion, k_out_, pulse secretion width, amount at time point 0 and pulse amplitude were estimated as population parameters. Population parameters were estimated with low relative standard errors (ranging from 2 to 5%). Total body water (%) was identified as a covariate for pulse amplitude, baseline secretion and the pulse secretion width following a power relationship. Simulations visualized multiple gradients of a hypothetical drug that influenced the endogenous secretion of GH. The established model was able to fit and quantify the highly variable individual 24 h GH profiles over time. This pharmacodynamic model can be used to quantify drug effects that target other endogenous pulsatile compounds.

**Electronic supplementary material:**

The online version of this article (doi:10.1007/s10928-017-9526-0) contains supplementary material, which is available to authorized users.

## Introduction

Many drugs exhibit wanted and/or unwanted effects on the secretion of endogenous pituitary hormones such as growth hormone (GH), prolactin or luteinizing hormone (LH). These hormones are secreted in ‘bursts’ or pulses and, particularly in the case of GH, vary in amplitude, time of secretion and may be influenced by circadian rhythmicity and sleep [1–4]. When studying drug effects on the secretion of GH, a non-compartmental analysis on the plasma concentration–time profile is commonly performed. This results in mean and maximum plasma GH concentrations over a specified time interval or the area under the plasma GH concentration–time curve (AUC) to be used for comparison between groups [5–7]. Importantly, by reducing the complex concentration–time profile of GH to summary statistics, the use of time as a continuous variable is lost. Therefore, the analysis results are highly dependent on the timeframe of observations and sampling interval, e.g. the endogenous GH AUC of 0–12 h can yield different results than the AUC of 12–24 h within the same individual due to the secretion of variable pulses. This contributes to high variability in these summary statistics. Furthermore, the commonly used single GH measurement or multiple-point mean [8, 9] does not capture the total secretion profile of an individual and thereby limits the correct quantification of a possible drug effect over time.

More advanced analysis methods, such as deconvolution analysis, have been developed to extract more information from a pulsatile profile [10, 11]. With deconvolution analysis, the observed concentrations are treated as the product of secretion and elimination processes. The underlying pulsatile secretion processes are estimated as Gaussian shaped events. The time points of these events are optimized to fit the data via multiple in- and exclusion steps [3, 10, 12]. Deconvolution analysis provides information on the regularity, the frequency, the amplitude, baseline secretion and the secretion width of pulses on an individual level [12, 13]. Even though this increases the amount of information that is retrieved from pulsatile GH concentration–time profiles, time cannot be used as a continuous variable in the reported tables. Thus, deconvolution results are still dependent on the study design, and it therefore has limited utility in comparing results between studies where different dosing regimens or study designs are used.

In drug development, population approach non-linear mixed effects (NLME) models are often used to study the pharmacokinetics and pharmacodynamics (PK/PD) of drugs over time. For example, direct effect, turnover or pool models are commonly used to describe endogenous pituitary hormone secretion over time [14–16]. However, such pharmacodynamic models cannot account for a highly variable pulsatile secretion. In the literature, the implementation of pulsatile functions in NLME models has been limited to compounds with a low number of pulses (melatonin [17], LH [2], ACTH [18]). When a higher number of pulses is observed, the numerical complexity of the model increases and the model stability decreases when the pulse location, duration and amplitude of multiple pulses need to be estimated.

The aim of this study is to develop a new method to quantify and model pulsatile data for the development of a pharmacodynamic model that is able to quantify highly variable pulsatile secretion patterns over time by combining deconvolution analysis techniques and NLME modeling. Deconvolution analysis was previously performed on data from a clinical study where GH concentrations were sampled every 10 min over a 24 h period in normal weight, upper body obese (UBO) women with large visceral fat areas and lower body obese (LBO) women with small visceral fat areas, before and after weight loss [19]. This resulted in the identification of reduced GH secretion in UBO subjects and no significant difference in GH half-life or volume of distribution. This densely sampled and highly variable dataset was used for the model development of this study. The deconvolution method used in the study of Pijl et al. [19] has been improved and applied in a new software package, AutoDecon, which was implemented in this study [3]. Furthermore, GH concentration–time profiles after the administration of hypothetical drugs that have antagonistic or agonistic properties were simulated. As a proof of concept of the application of this method on the quantification of a drug effect, a simulated drug effect was re-estimated using data from a clinical trial simulation.

## Methods

### Study design

Data were obtained from a clinical study which has been reported previously [19]. In short, 16 women (8 UBO and 8 LBO subjects) followed a weight loss diet to study GH kinetics, before and after weight loss, compared to normal weight control subjects (N = 8). Blood samples for GH analysis were taken at 10 min intervals for 24 h, resulting in a maximum of 144 samples per individual per occasion. The lower limit of quantification (LLOQ) for the GH immunofluorometric assay was 0.03 mU/L.

### Deconvolution analysis

Individual deconvolution analysis of the 24 h GH concentration time profiles was performed in AutoDecon [3]. This software provides a “nonsubjective, standardized, and completely automated algorithm” [3] for the analysis of pulsatile profiles. During data assembly, the expected measurement times with 10 min intervals were used, as this program requires regularly spaced time input. Separate data files were created for each individual in which the mean GH observation of the duplicate measurement, the standard error of the mean and the number of analysis replicates for each time point were included. The previously reported coefficient of variation of the observations over the concentration range was used to calculate the standard error of the mean for each observation [10, 19]. For AutoDecon to function, initial estimates of the secretion width and an initial half-life of GH (5 and 15 min, respectively) were used as input. These parameters were optimized for each individual during the deconvolution analysis. The convolution integral that is implemented in AutoDecon is depicted in Eq. 1 [3].

1 $$C\left( t \right) = \mathop \smallint \limits_{0}^{t} S_{n} \left( \tau \right)E\left( {t - \tau } \right)d\tau + C\left( 0 \right)E(t)$$

where *C*(*t*) is the GH concentration over time, consisting of the integral of all secretion events (*S*_*n*_(*τ*)), plus the concentration at time point zero (*C*(*0*)). The elimination function (*E*) can follow a 1- or 2-compartment disposition for GH, determined in AutoDecon.

Deconvolution analysis resulted in the estimation of an individual’s pulse frequency, pulse times, secretion width, baseline secretion, pulse amplitude and half-life. After deconvolution analysis, statistical comparison of the results between the LBO and UBO groups compared to normal weight control subjects was done using an independent 2-group *t* test.

### Model development

For each individual, the pulse frequency and the individual pulse times, retrieved from the deconvolution analysis, were included in the dataset for NLME modeling (NONMEM V7.3 [20]). The random effects structure was incorporated in the model by a ln-normal transformation of the random effects (*η*) on the population parameters [21]. Significant inter-individual variability (IIV) on population parameters was included in the model following a forward inclusion method (p < 0.05). The residual error distribution (*ε*) was drawn using an additive, proportional or combined (additive and proportional) residual error structure using parameters from a normal distribution. Various types of variance–covariance matrices were tested for the correlation between the random effects when identified by Pearson correlation plots. Due to high IIV and intra-individual variability in the height of the pulses (pulse amplitude) within a 24 h concentration–time profile, each pulse was estimated as a different occasion (BOV) which enabled the estimation of GH pulses of different heights within one individual. The amplitude of a pulse was modeled according to Eq. 2.

2 $$Amplitude_{n} = \theta_{\text{population}} \cdot {\text{e}}^{{\left( {\eta + \kappa_{n} } \right)}}$$

where *Amplitude*_*n*_ is the amplitude of pulse *n*, *θ*_*population*_ is the population amplitude parameter, *η* is the random effects distribution of the IIV and *κ*_*n*_ is the BOV, the intra-individual variability, for pulse *n*.

Two equations (Eqs. 3, 4) were tested to fit a pulsatile event in the NLME model. Equation 3 is adapted from the documentation of AutoDecon [3], Eq. 4 is adapted from a previous publication that models a single Gaussian shaped pulse in NONMEM [2].

3 $$S_{n} ({\text{t}}) = {\text{e}}^{{\ln \left( {{\text{Amplitude}}_{n} } \right) - \frac{1}{2} \cdot \left( {\frac{{{\text{t}} - {\text{PulseTime}}_{n} }}{\text{SecretionSD}}} \right)^{2} }}$$

4 $$S_{n} ({\text{t}}) = \frac{{{\text{Amplitude}}_{n} }}{{\left( {\frac{{{\text{t}} - {\text{PulseTime}}_{n} }}{\text{SecretionSD}}} \right)^{\text{exponent}} + 1}}$$

where *S*_*n*_(*t*) is the secretion over time for pulse *n*, *Amplitude*_*n*_ is the amplitude of pulse *n*, *PulseTime*_*n*_ is the time which corresponds with the maximum secretion time for pulse *n* (retrieved from deconvolution analysis), and *SecretionSD* corresponds with the width of the pulses. In Eq. 3, the exponential transformation limits the *S*_*n*_(*t*) to positive values only. In Eq. 4, the exponents 2 and 4 were evaluated during model development.

### Covariates

The following covariates were explored: weight, height, age, lean body mass (LBM), total body water (TBW), total body fat, percentage fat mass, percentage LBM and percentage TBW. All weight related covariates were calculated using Bioelectrical Impedance Analysis (Bodystat 1500, Bodystat Ltd., Isle of Man, UK). Covariate relationships were explored using visual exploration of the Pearson correlation plots and their correlation coefficients. When a correlation coefficient was 0.5 or higher, covariate relationships were formally tested for significance in the structural model using linear, power and exponential relationships. Circadian rhythmicity was explored as a covariate on the *Amplitude* parameter using a cosine function with a 24 h acrophase or as a day/night effect. Covariates were included using a forward inclusion method (p < 0.05) combined with backward elimination (p < 0.01) and centered around their mean values.

### Model evaluation

Models were evaluated on basis of objective function value (OFV, which approximates −2*Log Likelihood), goodness of fit (GOF) plots and numerical evaluation [22]. Model hypothesis testing was done under the assumption that the difference in OFV is χ^2^-distributed with the degrees of freedom determined by the number of additional parameters in the more complex model. A drop in OFV of more than 3.84 points (p = 0.05) resulted in accepting the model with one additional degree of freedom. For backward elimination of a covariate, an increase of less than 6.6 points in OFV (p = 0.01) was needed for exclusion. Model comparison implementing Eqs. 3 and 4 was done using the Bayesian information criterion (BIC) due to changes in the structural model. GOF plots consisted of population- and individual model predictions versus observations, conditional weighted residuals with interaction (CWRESI) versus clock time and population predictions. Numerical evaluation was based on the relative standard error (RSE) for population parameters, normalized prediction distribution error (NPDE) analysis, coefficient of variation for random effects (CV%) and the condition number [22, 23].

### Simulation

Multiple simulations with the developed model were performed to visualize the effect of hypothetical drugs on the GH concentration–time profile targeting GH secretion. For these simulations, the parameter estimates of the developed model were used to simulate a typical individual with a pulse interval of 1.57 h (estimated mean pulse interval). The half-life of the hypothetical drug was fixed to 6 h to simulate a short-term effect which can still be observed in a 24 h period. The drug effect was implemented using an *E*_*max*_ relationship driven by the amount of the hypothetical drug where the maximum effect was reached immediately after bolus dose administration (10 mg). The effect of the drug on the pulsatile secretion was modeled using Eqs. 5 and 6.

5 $$Effect\left( t \right) = \frac{{E_{max } \cdot A(t)^{\gamma } }}{{EA_{50}^{\gamma } + A(t)^{\gamma } }}$$

6 $$S_{n} \left( t \right) = e^{{\ln \left( {Amplitude_{n} } \right) - \frac{1}{2} \cdot (\frac{{t - PulseTime_{n} }}{SecretionSD})^{2} }} \cdot(1 + Effect(t))$$

where *E*_*max*_ varied between −1 and 10 to simulate inhibitory and stimulatory effects and was 0 for simulations of the typical individual. *A*(*t*) is the amount of the hypothetical drug remaining in the body. For simulations of the inhibitory drug effect, the *E*_*max*_ was equal to −1, −0.75 and 0. For simulations of the stimulatory drug effect, the *E*_*max*_ was equal to 0, 2, 5 and 10. *EA*_*50*_ was fixed to 2 mg with *γ* equal to 5 to simulate a relatively fast offset of the drug effect within 18 h after dosing. Covariate relationships were simulated at their mean values.

A new clinical trial was simulated to investigate whether the applied drug effects could be correctly re-estimated using the developed method proposed in this study. Simulations were performed including the identified IIV and residual error structure of the developed model. Pulses were simulated at regular time intervals. Additionally, a CV% of 10% was added on the *EA*_*50*_. A total of 5 cohorts of 8 subjects were simulated (placebo, 2.5, 5, 7.5 and 10 mg) with an *E*_*max*_ of −0.9 (inhibiting the GH secretion by 90%) and an *EA*_*50*_ of 3 mg with *γ* equal to 5. The parameters estimated in the deconvolution-analysis-informed population model were fixed to their corresponding values. The re-estimated drug effect parameters were then compared to the simulated ‘true’ model values to judge the ability to correctly recover the drug effect given this true model.

### Software

All data transformations, statistical tests on the deconvolution results, visualizations and NPDE analysis were performed using R (V3.2.2) [24] in conjunction with R Studio (V0.99.887) [25]. AutoDecon (V20090124) [3] was used for the deconvolution analysis of individual 24 h GH profiles. NLME modeling was performed in NONMEM V7.3 [20]. The BIC was calculated using Pirana (V2.9.0) [26], no changes to the default BIC calculations were made.

## Results

**Fig. 1:**
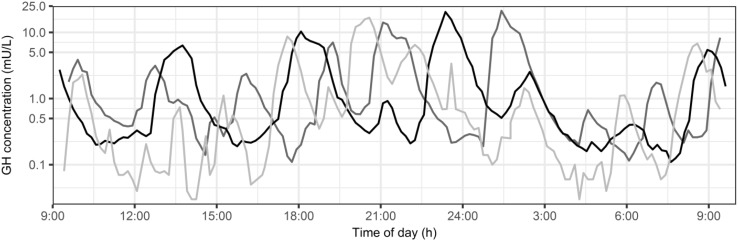
Observed growth hormone concentrations of three individuals over time of day

A total of 2 subjects (1 from the LBO and 1 from the UBO group) did not complete the occasion after weight loss. For these individuals, only data from the visit before weight loss were included in model development. Data below the LLOQ (n = 52, <1%) were excluded from this analysis. A total of 5377 GH observations were used for model development. Figure 1 visualizes the GH concentration–time profiles during the 24 h observation period for three individuals. High intra- and inter-individual variability can be observed in the pulse amplitude and the time of GH secretion.

### Deconvolution

Table 1 shows the summary of the deconvolution analysis results for each group, before and after weight loss. A 1-compartment elimination function was identified as the best fit for GH disposition. The secretion width of the pulses was found to be significantly lower in LBO subjects before weight loss and in all UBO subjects compared to normal weight subjects. The UBO subjects before weight loss showed a reduction in the baseline secretion compared to normal weight subjects. No significant differences in the pulse frequency, half-life, amplitude or pulse interval were identified between normal weight and obese subjects.

**Table 1:** Deconvolution analysis results reported as mean (sd), estimated by AutoDecon

Parameter	Normal weight (n = 8)	LBO		UBO	
		Before WL (n = 8)	After WL (n = 7)	Before WL (n = 8)	After WL (n = 7)
Pulse frequency^a^	15 (4)	17.8 (4.8)	17.4 (5.4)	14.6 (3.4)	16.4 (5.4)
Half-life (h)	0.245 (0.065)	0.233 (0.032)	0.26 (0.033)	0.235 (0.033)	0.247 (0.042)
Secretion width (h)	0.55 (0.118)	0.37 (0.168)*	0.44 (0.115)	0.37 (0.063)*	0.43 (0.086)*
Baseline secretion (mU/L/h)	0.666 (0.402)	0.636 (0.666)	0.738 (0.276)	0.288 (0.216)*	0.516 (0.372)
Amplitude	0.456 (0.234)	0.389 (0.227)	0.432 (0.132)	0.264 (0.202)	0.526 (0.337)
Pulse interval (h)	1.63 (0.49)	1.42 (0.54)	1.38 (0.38)	1.60 (0.42)	1.83 (1.45)

*WL* weight loss; *LBO* lower body obese; *UBO* upper body obese

^a^Total number of pulses in the 24 h period. * p < 0.05 between normal weight and LBO/UBO group

### Model development

**Fig. 2:**
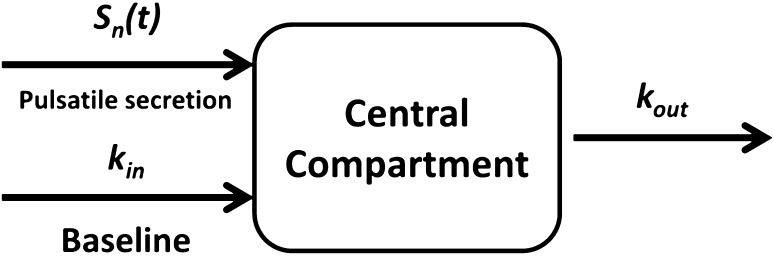
Structural model including a zero-order baseline (*k*_*in*_) and pulsatile secretion input (*S*_*n*_(*t*)) with a first-order elimination rate (*k*_*out*_)

The GH observations were best described using a turnover compartment, as depicted in Fig. 2. The baseline secretion was modeled as a steady-state condition, using a continuous zero-order input (*k*_*in*_) in the central compartment and first-order elimination (*k*_*out*_) that describes the elimination of GH from the body. The *k*_*in*_ was estimated as *Baseline · k*_*out*_ so that the *Baseline* parameter is estimated as mU/L. The pulsatile secretion is the sum of the secretion of all pulses at a certain time point (Eq. 7) where *npulses* is the pulse frequency of an individual. The differential equation of the central GH compartment is presented in Eq. 8.

7 $$S\left( t \right) = S_{1} \left( t \right) + S_{2} \left( t \right) + \ldots + S_{npulses} \left( t \right)$$

8 $$\frac{d(GH)}{dt} = k_{in} + S(t) - k_{out} \cdot GH$$

A large proportion of the subjects showed GH concentrations above baseline at the start of the measurement period (e.g. Figure 1, black line) due to the secretion of an endogenous pulse of GH prior to the start of the observation period. To account for these initial concentrations, the central compartment was initialized at an initial concentration (*A_0*). The estimation of BOV between the GH profiles before and after weight loss within one individual resulted in numerical difficulties due to the large number of random effects (54+) on the amplitude parameter to be estimated. Therefore, the two occasions of one individual were stratified using unique subject identifiers. Thereby reducing the number of random effects within one individual but losing the ability to identify intra-individual variability between occasions before and after weight loss.

IIV was identified on, in order of inclusion, *Baseline* (ΔOFV = −3702), *A_0* (ΔOFV = −1611), *k*_*out*_ (ΔOFV = −642.0), *SecretionSD* (ΔOFV = −419) and *Amplitude* (ΔOFV = −29). A 2 × 2 omega block was included after covariate analysis to account for variance–covariance correlation between the *Baseline* and *Amplitude*. A proportional residual error structure was best fit for purpose. The model fit was significantly better when using Eq. 3 (BIC = −3521.957) compared to the use of Eq. 4, where exponent = 4 (BIC = −2772.223) or exponent = 2 (BIC = −1619.14).

### Covariate analysis

**Fig. 3:**
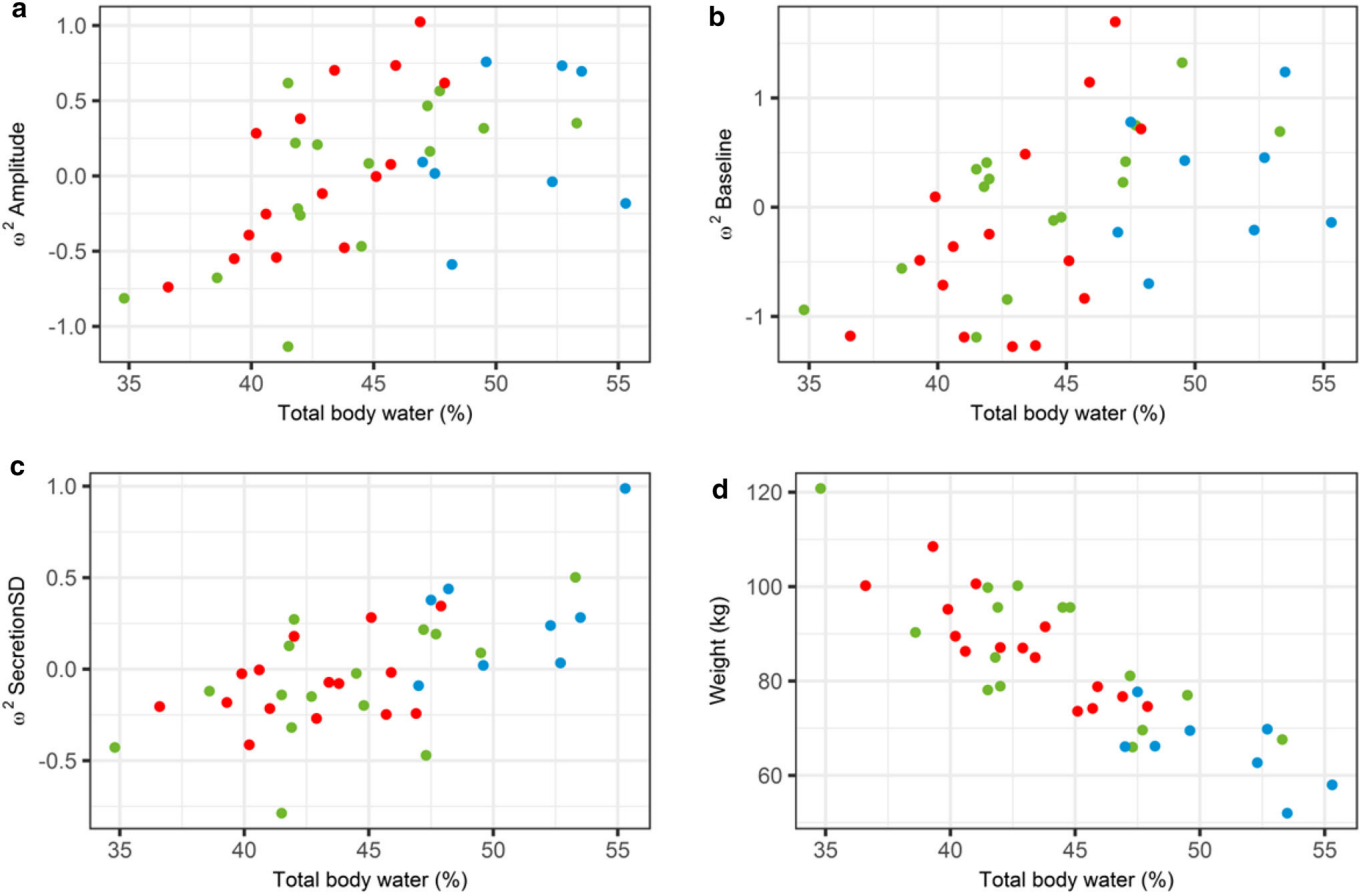
Correlation plots of individual ω^2^ estimates (*solid colored circles*) of **a** *Amplitude,* **b** *Baseline*, **c** *SecretionSD* and **d** weight (kg) versus the total body water (%). *Blue* normal weight subjects; *green* lower body obese subjects; *red* upper body obese subjects (Color figure online)

The estimation of a 24 h cosine function or a day/night effect on *Amplitude* did not result in the improvement of the OFV, indicating that no circadian rhythmicity could be identified on this data. Inspection of the correlation plots of the *ω* ^*2*^ distribution identified covariate relationships between the distribution of *Baseline*, *Amplitude* and *SecretionSD* with the TBW (%), as shown in Fig. 3. The TBW (%) also showed a high degree of correlation between the weight of a subject in which heavier subjects had a lower percentage total body water. A power covariate relationship showed to be superior over other tested relationships. The inclusion of this covariate relationship on the *Baseline*, *Amplitude* and *SecretionSD* parameters resulted in a significant decrease (ΔOFV = −48) in OFV. Backward elimination did not result in the removal of a covariate in this model. The previously observed covariate correlations between the random effects were reduced to a random scatter around 0 after inclusion of the covariate, indicating that part of the identified variability could be explained by the included covariate. The correlation plots of TBW(%) with the ω^2^ distribution of *Baseline*, *Amplitude* and *SecretionSD* after inclusion of the covariate relationship in the structural model, can be found in online resource I.

### Model evaluation

**Fig. 4:**
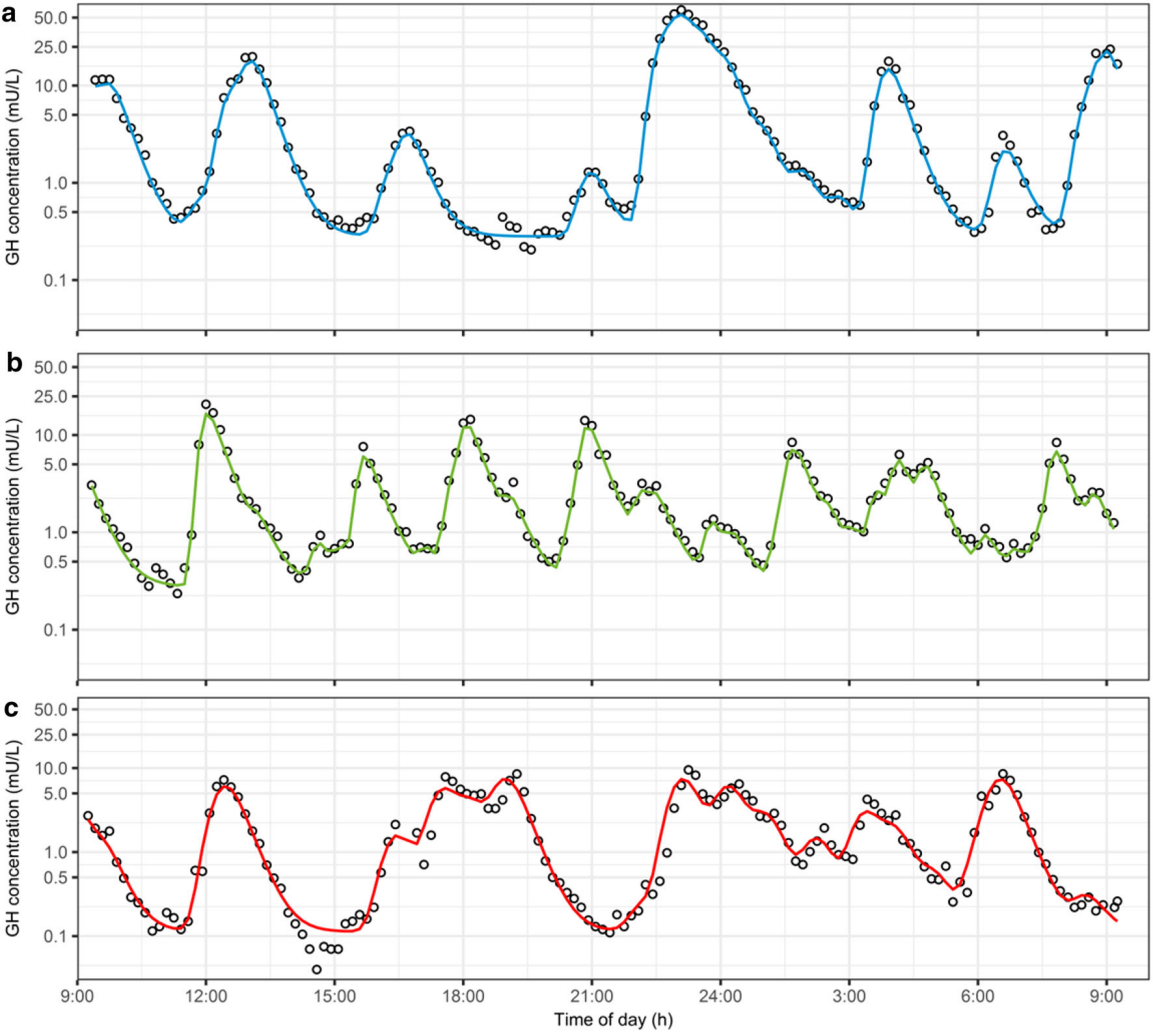
GH observations (mU/L) versus individual GH predictions of three individuals: **a** normal weight, **b** lower body obese and **c** upper body obese subject on a semi-logarithmic scale. *Black open dots*: observations, *solid colored line*: individual model predictions (Color figure online)

**Fig. 5:**
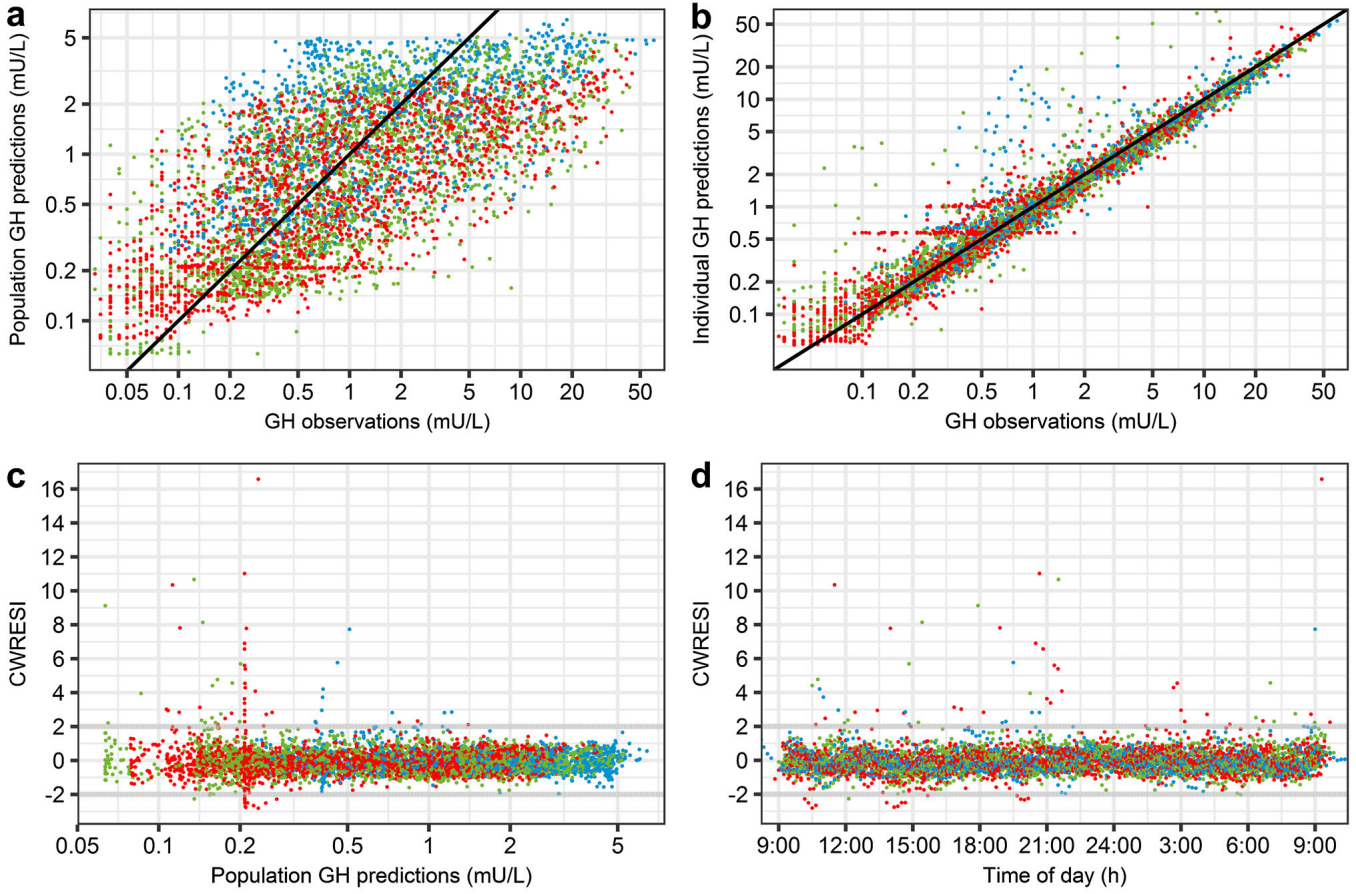
Goodness of fit plots for the deconvolution-analysis-informed population model. **a** Population GH model predictions versus observations **b** Individual GH model predictions versus observations **c** CWRESI versus population predictions **d** CWRESI versus time of day. *Blue* normal weight subjects; *green* lower body obese subjects; *red* upper body obese subjects. *Black diagonal line* indicates line of unity. *Grey dashed horizontal lines* indicate the [**−**2,2] interval (Color figure online)

For visualization purposes, individual model fits over time, for one individual per group, are depicted in Fig. 4. The model predictions clearly show the adequate model fit of the highly variable individual GH profiles in these individuals over a 24 h period. The individual tendency of all data is well described, with observations close to line of unity (individual observations vs. individual model predictions, Fig. 5). The individual observations vs. population model predictions show a broad scatter around the line of unity indicating an appropriate structural model combined with high variability between and within individuals. The CWRESI are normally distributed over the entire range of population predictions and the majority of the observations lie within the [−2,2] interval. Outliers identified in the CWRESI plots are resulting from mispredictions of pulse times by the deconvolution analysis. The highest CWRESI point results from the deconvolution analysis not being able to fit a pulse at the last time points where no information on the downward profile of the concentration is available. The parameter estimates of the final model are reported in Table 2. The population parameters show low RSEs (ranging from 2 to 5%), indicating high accuracy in the estimation of the population parameters. The CV% was moderate ranging from 26.9 to 70.8% for most population parameters. High CV% was identified for the *A_0* and the *κ* on *Amplitude* with a CV% of 521 and 302% respectively. The high CV% for these parameters originates from the differences in initial concentration (the possible occurrence of a pulse before the start of the observation period) and the high variability in pulse amplitudes between the separate pulses within one individual. The condition number of the final model was 11.3, indicating no ill conditioning [23]. NPDE analysis results can be found in online resource II. The NONMEM code of the final deconvolution-analysis-informed population model (FOCE+I, ADVAN = 13, TOL = 6, SIGL = 6, NSIG = 3) can be found in online resource III.

**Table 2:** Parameter estimates for the deconvolution-analysis-informed population model

Parameter	Unit	Estimate [RSE%] (CV%)	Shrinkage (%)
ϴ *Amplitude*	mU/L/44.7% TBW	7.86 [3.25]	–
ϴ *k*_*out*_	/h	2.78 [3.46]	–
ϴ *SecretionSD*	h/44.7% TBW	0.182 [3.14]	–
ϴ *Baseline*	mU/L/44.7% TBW	0.185 [4.52]	–
ϴ *A_0*	mU/L	1.05 [5.03]	–
ϴ Exponent *Amplitude*	–	3.4 [2.1]	–
ϴ Exponent *SecretionSD*	–	2.32 [3.15]	–
ϴ Exponent *Baseline*	–	4.29 [4.29]	–
ω^2^ *Amplitude*_*IIV*_	–	0.22 (49.7)	12.8
ω^2^ *Amplitude*_*BOV*-*n*_	–	2.32 (302)	–
ω^2^ *k*_*out*_	–	0.0699 (26.9)	2.02
ω^2^ *SecretionSD*	–	0.0715 (27.2)	0.12
ω^2^ *Baseline*	–	0.406 (70.8)	<0.01
ω^2^ *A_0*	–	3.34 (521)	0.18
σ^2^ proportional residual error	–	0.106	5.61

*RSE%* relative standard error; *CV%* coefficient of variation; *TBW* total body water

### Simulation

**Fig. 6:**
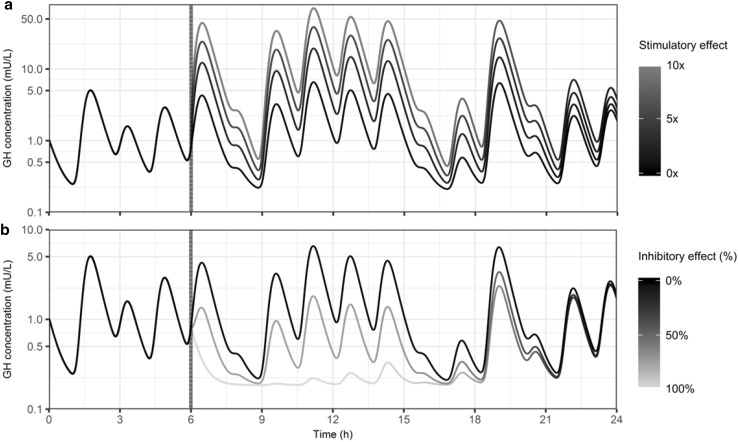
Simulated growth hormone profiles after administration of an agonistic (**a**) or antagonistic (**b**) hypothetical drug. *Dashed black vertical line* is the time of drug administration. *Color* gradient shows the drug effect over time returning back to normal (*black solid line*) (Color figure online)

The simulated GH concentration–time profiles are depicted in Fig. 6 and stratified on stimulatory (Fig. 6a) and inhibitory (Fig. 6b) drug effects. The hypothetical drug was administered after 6 h, indicated by the vertical dashed black line. Simulations of the typical individual (black line, 0×/0% effect) correspond with observed GH concentrations in terms of pulse amplitude, pulse width and baseline GH secretion for an individual with the mean TBW(%) of 44.7%. Stimulating the GH secretion showed a clear increase in the GH concentrations which decreased back to normal as can be seen by the color gradient. Inhibition of the GH secretion by 100% reduced the GH concentration back to baseline for the first hours after dosing. Thereafter, the GH secretion returned back to a pulsatile profile. The inhibition of GH secretion by 75% showed reduced GH concentrations but did not completely counter the pulsatile secretion of GH in this scenario. The drug effect over time is depicted in online resource IV.

The simulated clinical trial resulted in a total of 5800 observations which were then fitted to the true simulated model structure (including the drug effect) and to a reduced model structure (excluding the drug effect). Modeling the GH secretion while excluding a drug effect resulted in an OFV of −1871 points. The inclusion of an *E*_*max*_ relationship for the drug effect resulted in a significant drop in OFV (ΔOFV = −234). The re-estimated model parameters are presented in Table 3 and are near identical to the parameter values used for simulation of the clinical trial data. The low RSE indicates high accuracy in the re-estimation of the drug effect parameters given the true simulated model. High shrinkage (51%) was observed on the random effects of the *EA*_*50*_. The NONMEM model codes used for simulation and re-estimation, including the GOF plots for the re-estimated model, are provided in online resource V.

**Table 3:** Parameter estimates of the simulated and the re-estimated model

Parameter	Simulated	Re-estimated parameter estimates [RSE%] (CV%)	Shrinkage (%)
ϴ *E*_*max*_	−0.9	-0.929 [1.6]	–
ϴ *EA*_*50*_	3	3.16 [7.4]	–
ϴ *γ*	5	5.04 [21]	–
ω^2^ *EA*_*50*_	0.01	0.0363 (19.2)	51
σ^2^ proportional residual error	0.106	0.105	5.08

*RSE%* relative standard error; *CV%* coefficient of variation

## Discussion

The developed method, using NLME modeling with NONMEM, has proven to adequately describe variable endogenous pulsatile GH concentration–time profiles in women. This resulted in the identification of three covariate relationships and the quantification of BOV and IIV on the amplitude of pulses. In addition, simulations have been performed to show hypothetical drug effects on GH concentration–time profiles. The correct re-estimation of the drug effect in the simulated clinical trial data shows the applicability of this method in the analysis of variable pulsatile data.

The RSEs of the population parameters were low (<10%), indicating stable predictions of population parameters. The CV% on *A_0* and *Amplitude* were high, which is in agreement with the observed high variability in the observed concentration at time point 0 and between the amplitude of pulses within an individual, respectively. The high GH concentrations at the first measurement indicate that sampling should be performed at several pre-dose time points to be able to quantify GH pulses that may have occurred before dose administration. The population half-life of GH was estimated as 15 min (*ln*(2)*/k*_*out*_) in this population and can be compared with the half-life reported in literature [27–29]. No circadian rhythmicity was identified in this population. This could be due to potential differences between men and women in regards to nocturnal GH secretion [30].

The study of Pijl et al. [19] resulted in the identification of a stratification between the GH secretion in UBO compared to LBO and normal weight subjects. The deconvolution analysis performed in this study did also result in the identification of a lower baseline secretion in UBO subjects before weight loss. No changes in the pulse amplitude were identified between groups. In this study, significant continuous covariate relationships have been identified in which the total body water was the best covariate explaining the IIV for *Baseline*, *Amplitude* and *SecretionSD*. Furthermore, the possibility to follow the GH concentration over time in a NLME model increased the information that is retrieved from these dense sampled observations.

During model development, it was required to stratify the two occasions, before and after weight loss, of one individual with unique identifiers to prevent numerical instability of the model. This resulted in the loss of estimation of the intra-individual variability between these separate occasions. Due to the high variability in the GH profiles that were observed between the occasions before and after weight loss, the long duration between the occasions (up to 6 months) and the nature of this paper to establish a new quantification method using NLME modeling, this stratification was deemed appropriate.

The Gaussian shaped events that are fitted in deconvolution analysis have the advantage to be applicable on a wide range of pulsatile compounds [31, 32]. However, this generalization inherently brings the disadvantage that no mechanistic information of the biological regulation of the compound is incorporated in the structural model. Especially when multiple components are involved in the regulation of the secretion, e.g. stimulation by growth hormone releasing hormone and inhibition by somatostatin in the case of GH, more mechanistic insights can be beneficial in explaining the variability between individuals and possible extrapolation to disease states.

Compared to previously used non-compartmental analysis, the current analysis enables the quantification of a drug effect targeting GH secretion over time in which an *E*_*max*_ or other PK/PD relationships can be determined using NLME in NONMEM. The correct estimation of the PK/PD relationship parameters on a pulsatile compound will be mainly dependent on 3 components: (1) the frequency of sampling, which is critical for compounds with a short half-life [11], (2) the duration of the observation period should preferably include the on- and offset of the drug effect and (3) the expected variability in response should be taken into account when choosing the study sample size.

The method proposed in this study can be applied to analyze or simulate different pulsatile profiles. From the analysis step, information can be obtained on the pulsatile behavior (pulse frequency, width and amplitude) in a population and on the PK/PD relationship. From a clinical perspective, this provides more insight in the relationship between the pulsatile secretion of a compound and the effect that a drug has over time compared to differences in the AUC or C_max_ when comparing placebo with treated individuals. The provided simulations in this study visualize what effect hypothetical drugs can have on a pulsatile profile. This was simulated for drugs having agonistic or antagonistic properties. After administration of an agonistic compound with an *E*_*max*_ of 10, the maximum GH concentration reached was ~50 mU/L. These simulations are physiologically plausible since the simulated concentration are in agreement with data from acromegalic patients in which GH concentrations are in the same range [33]. The provided antagonistic simulations suggest a reduction to baseline after fully blocking the pulsatile secretion (*E*_*max*_ = −1). These antagonistic drug simulations (Fig. 6b) correspond with the clinical response as shown after treatment with Ocreotide, a somatostatin analogue, which can completely block the endogenous pulsatile secretion of GH for 5 h after dosing [7]. Blocking the pulse amplitude by 75% lowers the pulsatile GH secretion but did not fully block the pulsatile profile. The pulse interval can be changed in the model code to simulate a different pulse frequency, including variability on the pulse interval. Simulations incorporating the established *E*_*max*_ and/or *EC*_*50*_ values of a known drug on GH secretion can then be used to support dose selection or to investigate novel dosing regimens for the studied drug. The observation period of a study can be expanded if simulations show that the full effect is reached at a later stage than expected (e.g. indirect response or delayed release formulation of the drug) or that a fast offset of the drug effect suggests the need for more selective monitoring for multiple hours at an early stage compared to a full 24 h period.

Instead of using ‘simple’ summary statistics (mean, AUC, C_max_) for complex and variable pulsatile profiles, it is now possible to quantify and model a pulsatile profile over time and to identify what effect a potential drug has on the secretion. This two-step deconvolution-analysis-informed population pharmacodynamic model enables the possibility to analyze highly variable pulsatile profiles with a NLME PK/PD model in NONMEM.

## Electronic supplementary material

Below is the link to the electronic supplementary material.

Online resource 1 (DOCX 102 kb)

Online resource 2 (DOCX 125 kb)

Online resource 3 (DOCX 15 kb)

Online resource 4 (DOCX 466 kb)

Online resource 5 (DOCX 406 kb)
