# Supplementary material for: A two-step deconvolution-analysis-informed population pharmacodynamic modeling approach for drugs targeting pulsatile endogenous compounds
Source: J Pharmacokinet Pharmacodyn. 2017 May 11;44(4):389–400. doi: 10.1007/s10928-017-9526-0 (PMC5514197; doi:10.1007/s10928-017-9526-0)
Supplement: Supplementary file 1 — Online resource 1 (DOCX 102 kb) [file 10928_2017_9526_MOESM1_ESM.docx]

**Online resource I – Correlation plots after inclusion of covariates**

**
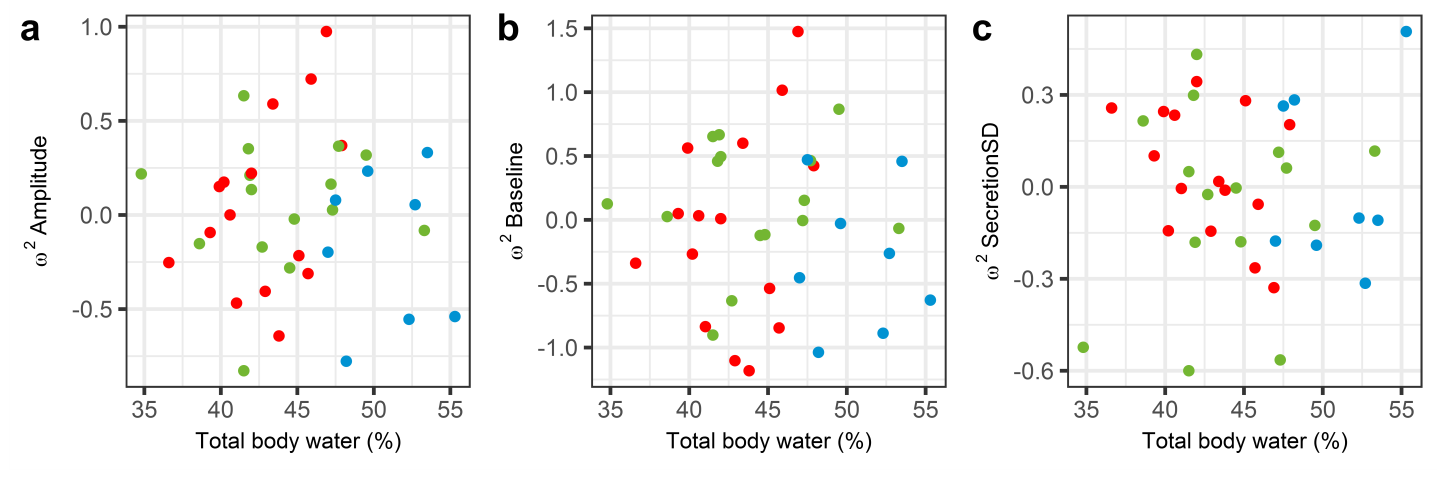
**

Correlation plots of individual ω^2^ estimates (solid colored circles) of a) *Amplitude*, b) *Baseline*, and c) *SecretionSD* versus the total body water (%) after inclusion of covariate relationships in the structural model. Blue: normal weight subjects, green: lower body obese subjects, red: upper body obese subjects.
