# Supplementary figures and images for: A two-step deconvolution-analysis-informed population pharmacodynamic modeling approach for drugs targeting pulsatile endogenous compounds
Source: J Pharmacokinet Pharmacodyn. 2017 May 11;44(4):389–400. doi: 10.1007/s10928-017-9526-0 (PMC5514197; doi:10.1007/s10928-017-9526-0)

**Online resource II – NPDE analysis results**

**
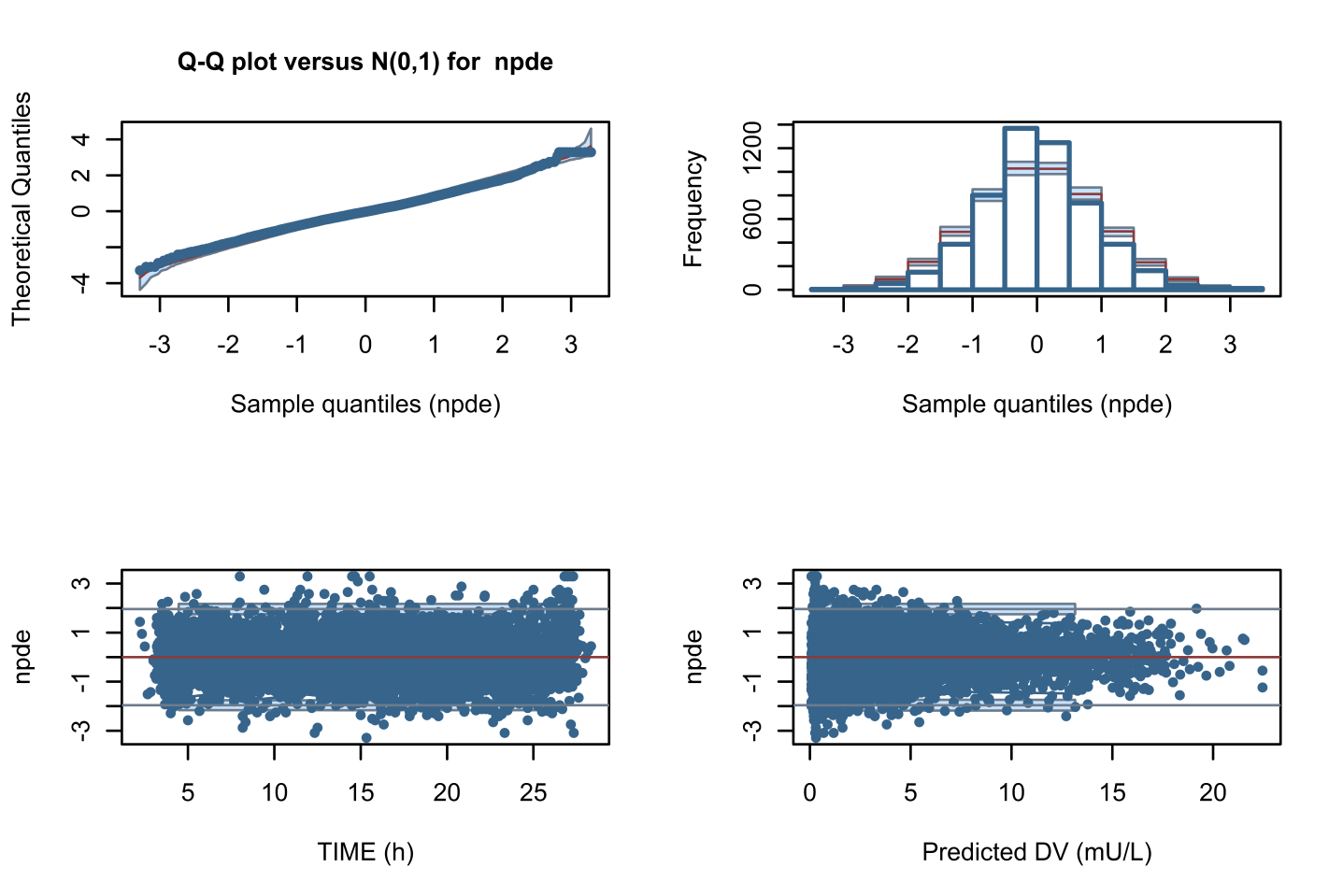
**

Supplement: Supplementary file 2 — Online resource 2 (DOCX 125 kb) [file 10928_2017_9526_MOESM2_ESM.docx]
