# Supplementary material for: A two-step deconvolution-analysis-informed population pharmacodynamic modeling approach for drugs targeting pulsatile endogenous compounds
Source: J Pharmacokinet Pharmacodyn. 2017 May 11;44(4):389–400. doi: 10.1007/s10928-017-9526-0 (PMC5514197; doi:10.1007/s10928-017-9526-0)
Supplement: Supplementary file 3 — Online resource 3 (DOCX 15 kb) [file 10928_2017_9526_MOESM3_ESM.docx]

**Online resource III – Model code (NONMEM V7.3)**

**$PROBLEM Deconvolution-analysis-informed pharmacodynamic model for growth hormone secretion**

**$INPUT ID TIME DV MDV WATERP FREQ P1:Pn**

**$DATA NONMEMDataset.csv IGNORE=I**

**$SUBS ADVAN=13 TOL=6**

**$MODEL**

**NCOMP=1**

**COMP=(DV,DEFDOS,DEFOBS)**

**$PK**

**KOUT = THETA(1)*EXP(ETA(1))**

**TVBase = THETA(2)*(WATERP/44.69)**THETA(6)**

**BASELINE = TVBase*EXP(ETA(2))**

**TVAmplitude = THETA(3)*(WATERP/44.69)**THETA(7)**

**IAMP = TVAmplitude*EXP(ETA(3))**

**A_0(1) = THETA(4)*EXP(ETA(4))**

**TVSecretionSD= THETA(5)*(WATERP/44.69)**THETA(8)**

**SecretionSD = TVSecretionSD*EXP(ETA(5))**

**KIN = KOUT*BASELINE**

**IF(FREQ.GE.1) AMPL1 = IAMP*EXP(ETA(6))**

**IF(FREQ.GE.2) AMPL2 = IAMP*EXP(ETA(7))**

**: ; Add separate line for each pulse**

**:**

**IF(FREQ.GE.n) AMPLn = IAMP*EXP(ETA(n))**

**$DES**

**RIN1 = 0+EXP(LOG(AMPL1)-0.5*((T-P1)/SecretionSD)**2)**

**RIN2 = 0+EXP(LOG(AMPL2)-0.5*((T-P2)/SecretionSD)**2)**

**: ; Add separate line for each pulse**

**:**

**RINn = 0+EXP(LOG(AMPLn)-0.5*((T-Pn)/SecretionSD)**2)**

**SECRETION = RIN1+RIN2+…+RINn**

**DADT(1)= KIN + SECRETION - KOUT*A(1)**

**$ERROR**

**IPRE = 0.00001**

**IF (F.GT.0) IPRE=F**

**Y=IPRE*(1+EPS(1))**

**$THETA**

**2.78 ; kout ( /h)**

**0.185 ; Baseline (mU/L)**

**7.86 ; Individual amplitude (mU/L)**

**1.05 ; A_0(1) (mU/L)**

**0.182 ; SecretionSD (h)**

**4.29 ; Exponent covariate relationship Baseline**

**3.4 ; Exponent covariate relationship Amplitude**

**2.32 ; Exponent covariate relationship SecretionSD**

**$OMEGA**

**0.0699 ; kout**

**$OMEGA BLOCK(2)**

**0.406 ; Baseline**

**0.233 0.22 ; Individual amplitude**

**$OMEGA**

**3.34 ; A_0**

**0.0715 ; SecretionSD**

**$OMEGA BLOCK(1) 2.32 ; Variability of amplitudes between pulses**

**$OMEGA BLOCK(1) SAME (n)**

**$SIGMA**

**0.106 ; Proportional residual error model**

**$EST PRINT=5 MAX=9999 METHOD=1 NSIG=3 SIGL=6 INTERACTION POSTHOC NOABORT MSFO=mfi**

**$COV PRINT=E**

**$TABLE ID TIME MDV DV WATERP CWRESI IPRE PRED RES WRES NOAPPEND NOPRINT ONEHEADER FILE=Modeloutput.table**
