# Supplementary material for: A two-step deconvolution-analysis-informed population pharmacodynamic modeling approach for drugs targeting pulsatile endogenous compounds
Source: J Pharmacokinet Pharmacodyn. 2017 May 11;44(4):389–400. doi: 10.1007/s10928-017-9526-0 (PMC5514197; doi:10.1007/s10928-017-9526-0)
Supplement: Supplementary file 4 — Online resource 4 (DOCX 466 kb) [file 10928_2017_9526_MOESM4_ESM.docx]

**Online resource IV – Simulations including drug effect over time**


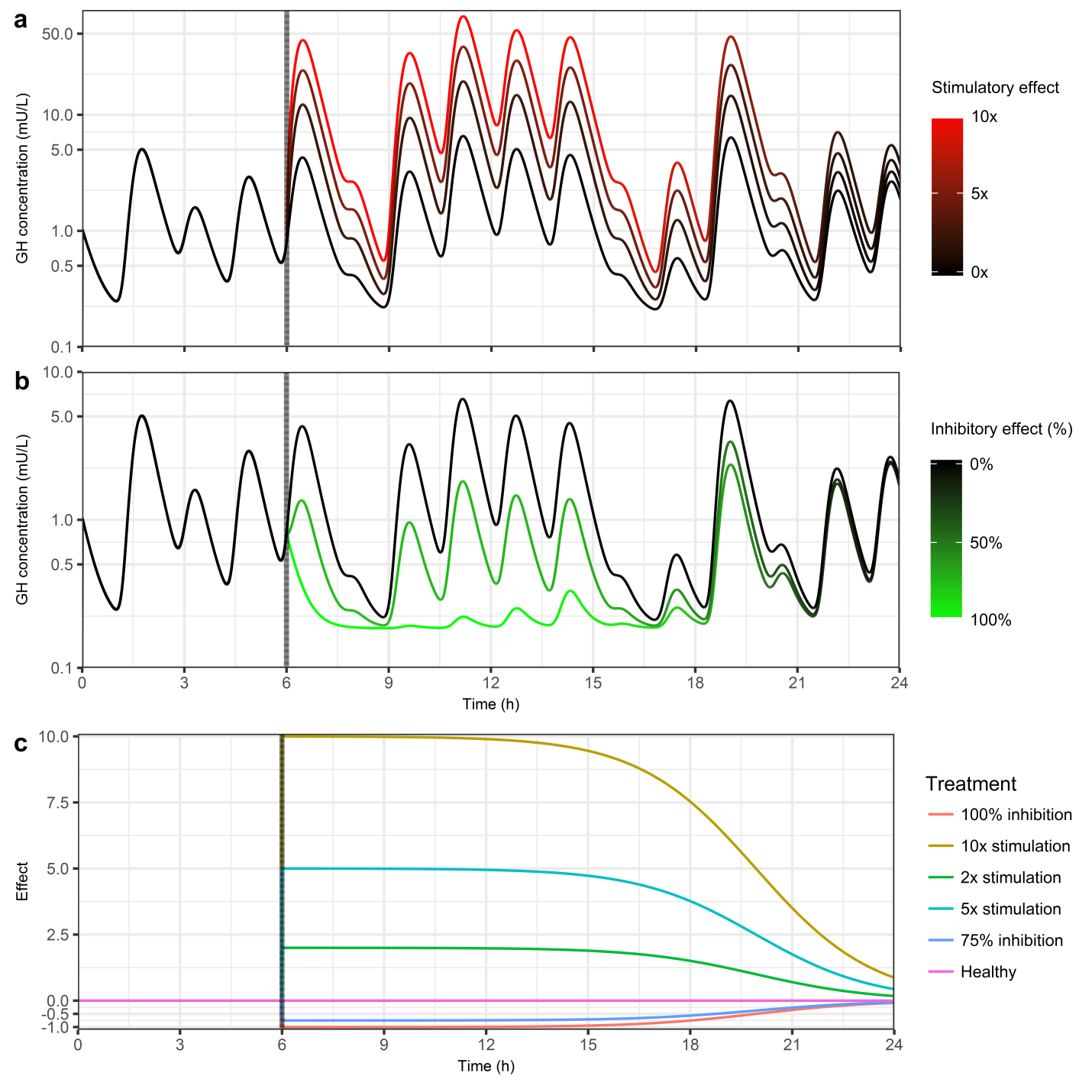


**Simulated growth hormone profiles after administration of an agonistic (a) or antagonistic (b) hypothetical drug. Dashed black vertical line is the time of dose administration. (c) shows the simulated drug effect over time. The color gradient shows the decrease of the drug effect over time back to normal (black solid line) in (a) and (b).**
