# Supplementary material for: A two-step deconvolution-analysis-informed population pharmacodynamic modeling approach for drugs targeting pulsatile endogenous compounds
Source: J Pharmacokinet Pharmacodyn. 2017 May 11;44(4):389–400. doi: 10.1007/s10928-017-9526-0 (PMC5514197; doi:10.1007/s10928-017-9526-0)
Supplement: Supplementary file 5 — Online resource 5 (DOCX 406 kb) [file 10928_2017_9526_MOESM5_ESM.docx]

**Online resource V – Simulation and re-estimation of drug effect**

***NONMEM model code – Simulation***

**$PROBLEM Simulation of clinical trial with 5 cohorts - inhibitory effect of 90%**

**$INPUT ID TIME DV AMT CMT MDV FREQ**

**$DATA Simulationdataset..csv IGNORE=I**

**$SUBS ADVAN=13 TOL=9**

**$MODEL**

**NCOMP=2**

**COMP=(GH)**

**COMP=(PK)**

**$PK**

**KOUT = THETA(1)*EXP(ETA(1))**

**; COV relation**

**WATERP=44.69329**

**TVBase = THETA(2)*(WATERP/44.69)**THETA(7)**

**BASELINE = TVBase*EXP(ETA(2))**

**TVAMP = THETA(3)*(WATERP/44.69)**THETA(6)**

**IAMP = TVAMP *EXP(ETA(3))**

**A_0(1) = THETA(4)*EXP(ETA(4))**

**TVSW = THETA(5)*(WATERP/44.69)**THETA(8)**

**SW = TVSW*EXP(ETA(5))**

**KIN = KOUT*BASELINE**

**;PD - Drug effect**

**kel = 0.11552453009**

**Emax = THETA(9) *EXP(ETA(26))**

**ec50 = THETA(10) *EXP(ETA(27))**

**gamma = THETA(11) *EXP(ETA(28))**

**IF(FREQ.GE.1) AMPL1 = IAMP*EXP(ETA(6))**

**IF(FREQ.GE.2) AMPL2 = IAMP*EXP(ETA(7))**

**IF(FREQ.GE.3) AMPL3 = IAMP*EXP(ETA(8))**

**IF(FREQ.GE.4) AMPL4 = IAMP*EXP(ETA(9))**

**IF(FREQ.GE.5) AMPL5 = IAMP*EXP(ETA(10))**

**IF(FREQ.GE.6) AMPL6 = IAMP*EXP(ETA(11))**

**IF(FREQ.GE.7) AMPL7 = IAMP*EXP(ETA(12))**

**IF(FREQ.GE.8) AMPL8 = IAMP*EXP(ETA(13))**

**IF(FREQ.GE.9) AMPL9 = IAMP*EXP(ETA(14))**

**IF(FREQ.GE.10) AMPL10 = IAMP*EXP(ETA(15))**

**IF(FREQ.GE.11) AMPL11 = IAMP*EXP(ETA(16))**

**IF(FREQ.GE.12) AMPL12 = IAMP*EXP(ETA(17))**

**IF(FREQ.GE.13) AMPL13 = IAMP*EXP(ETA(18))**

**IF(FREQ.GE.14) AMPL14 = IAMP*EXP(ETA(19))**

**IF(FREQ.GE.15) AMPL15 = IAMP*EXP(ETA(20))**

**IF(FREQ.GE.16) AMPL16 = IAMP*EXP(ETA(21))**

**IF(FREQ.GE.17) AMPL17 = IAMP*EXP(ETA(22))**

**IF(FREQ.GE.18) AMPL18 = IAMP*EXP(ETA(23))**

**IF(FREQ.GE.19) AMPL19 = IAMP*EXP(ETA(24))**

**IF(FREQ.GE.20) AMPL20 = IAMP*EXP(ETA(25))**

**S1=1**

**; Simulate PeakTimes**

**P1 = 1.57**

**P2 = P1 + 1.57**

**P3 = P2 + 1.57**

**P4 = P3 + 1.57**

**P5 = P4 + 1.57**

**P6 = P5 + 1.57**

**P7 = P6 + 1.57**

**P8 = P7 + 1.57**

**P9 = P8 + 1.57**

**P10 = P9 + 1.57**

**P11 = P10 + 1.57**

**P12 = P11 + 1.57**

**P13 = P12 + 1.57**

**P14 = P13 + 1.57**

**P15 = P14 + 1.57**

**P16 = P15 + 1.57**

**P17 = P16 + 1.57**

**P18 = P17 + 1.57**

**P19 = P18 + 1.57**

**P20 = P19 + 1.57**

**$DES**

**E= (Emax*(A(2)**gamma))/(ec50**gamma+A(2)**gamma)**

**RIN1 = 0+EXP(LOG(AMPL1)-0.5*((T-P1)/SW)**2)*(1-E)**

**RIN2 = 0+EXP(LOG(AMPL2)-0.5*((T-P2)/SW)**2)*(1-E)**

**RIN3 = 0+EXP(LOG(AMPL3)-0.5*((T-P3)/SW)**2)*(1-E)**

**RIN4 = 0+EXP(LOG(AMPL4)-0.5*((T-P4)/SW)**2)*(1-E)**

**RIN5 = 0+EXP(LOG(AMPL5)-0.5*((T-P5)/SW)**2)*(1-E)**

**RIN6 = 0+EXP(LOG(AMPL6)-0.5*((T-P6)/SW)**2)*(1-E)**

**RIN7 = 0+EXP(LOG(AMPL7)-0.5*((T-P7)/SW)**2)*(1-E)**

**RIN8 = 0+EXP(LOG(AMPL8)-0.5*((T-P8)/SW)**2)*(1-E)**

**RIN9 = 0+EXP(LOG(AMPL9)-0.5*((T-P9)/SW)**2)*(1-E)**

**RIN10 = 0+EXP(LOG(AMPL10)-0.5*((T-P10)/SW)**2)*(1-E)**

**RIN11 = 0+EXP(LOG(AMPL11)-0.5*((T-P11)/SW)**2)*(1-E)**

**RIN12 = 0+EXP(LOG(AMPL12)-0.5*((T-P12)/SW)**2)*(1-E)**

**RIN13 = 0+EXP(LOG(AMPL13)-0.5*((T-P13)/SW)**2)*(1-E)**

**RIN14 = 0+EXP(LOG(AMPL14)-0.5*((T-P14)/SW)**2)*(1-E)**

**RIN15 = 0+EXP(LOG(AMPL15)-0.5*((T-P15)/SW)**2)*(1-E)**

**RIN16 = 0+EXP(LOG(AMPL16)-0.5*((T-P16)/SW)**2)*(1-E)**

**RIN17 = 0+EXP(LOG(AMPL17)-0.5*((T-P17)/SW)**2)*(1-E)**

**RIN18 = 0+EXP(LOG(AMPL18)-0.5*((T-P18)/SW)**2)*(1-E)**

**RIN19 = 0+EXP(LOG(AMPL19)-0.5*((T-P19)/SW)**2)*(1-E)**

**RIN20 = 0+EXP(LOG(AMPL20)-0.5*((T-P20)/SW)**2)*(1-E)**

**SECRETION = RIN1+RIN2+RIN3+RIN4+RIN5+RIN6+RIN7+RIN8+RIN9+RIN10+RIN11+RIN12+RIN13+RIN14+RIN15+RIN16+RIN17+RIN18+RIN19+RIN20**

**DADT(1)= KIN + SECRETION - KOUT*A(1)**

**CGH = A(1)**

**DADT(2) = -Kel*A(2)**

**$ERROR**

**IPRE = 0.00001**

**IF (F.GT.0) IPRE=F**

**Y=IPRE*(1+EPS(1))**

**$THETA**

**2.78 FIX ; kout ( /h)**

**0.185 FIX ; Baseline (mU/L)**

**7.86 FIX ; Individual amplitude (mU/L)**

**1.05 FIX ; A_0(1) (mU/L)**

**0.182 FIX ; SecretionSD (h)**

**3.4 FIX ; Exponent covariate relationship Amplitude**

**4.29 FIX ; Exponent covariate relationship Baseline**

**2.32 FIX ; Exponent covariate relationship SecretionSD**

**0.9 FIX ; Emax**

**3 FIX ; EA50**

**5 FIX ; Gamma**

**$OMEGA**

**0.0699 FIX ; Kout**

**$OMEGA BLOCK(2)**

**0.406 ; Baseline**

**0.233 0.22 FIX ; Individual amplitude**

**$OMEGA**

**3.34 FIX ; A_0**

**0.0715 FIX; Secretion Width**

**$OMEGA BLOCK(1) 2.32 FIX; BOV on Amplitude 1-20**

**$OMEGA BLOCK(1) SAME (19)**

**$OMEGA**

**0 FIX ; EMAX**

**0.01 FIX ; EC50**

**0 FIX ; GAMMA**

**$SIGMA**

**0.106 FIX ; Proportional residual error model**

**$SIMULATION (123456) ONLYSIM SUBPROBLEMS=1**

**$TABLE ID TIME MDV AMT CMT CGH IAMP NOAPPEND NOPRINT ONEHEADER FILE=simulationmodel.res**

***NONMEM model code – Re-estimation***

**$PROBLEM Re-estimation of clinical trial data with 5 cohorts – inhibition of amplitude**

**$INPUT ID TIME MDV AMT CMT DV P1 P2 P3 P4 P5 P6 P7 P8 P9 P10 P11 P12 P13 P14 P15 P16 P17 P18 P19 P20 FREQ**

**$DATA SimulatedData.csv IGNORE=I**

**$SUBS ADVAN=13 TOL=9**

**$MODEL**

**NCOMP=2**

**COMP=(GH)**

**COMP=(PK)**

**$PK**

**KOUT = THETA(1)*EXP(ETA(1))**

**; COV relation**

**WATERP=44.69329**

**TVBase = THETA(2)*(WATERP/44.69)**THETA(7)**

**BASELINE = TVBase*EXP(ETA(2))**

**TVAMP = THETA(3)*(WATERP/44.69)**THETA(6)**

**IAMP = TVAMP *EXP(ETA(3))**

**A_0(1) = THETA(4)*EXP(ETA(4))**

**TVSW = THETA(5)*(WATERP/44.69)**THETA(8)**

**SW = TVSW*EXP(ETA(5))**

**KIN = KOUT*BASELINE**

**;PD - Drug effect**

**kel = 0.11552453009**

**Emax = THETA(9) *EXP(ETA(26))**

**ec50 = THETA(10) *EXP(ETA(27))**

**gamma = THETA(11) *EXP(ETA(28))**

**IF(FREQ.GE.1) AMPL1 = IAMP*EXP(ETA(6))**

**IF(FREQ.GE.2) AMPL2 = IAMP*EXP(ETA(7))**

**IF(FREQ.GE.3) AMPL3 = IAMP*EXP(ETA(8))**

**IF(FREQ.GE.4) AMPL4 = IAMP*EXP(ETA(9))**

**IF(FREQ.GE.5) AMPL5 = IAMP*EXP(ETA(10))**

**IF(FREQ.GE.6) AMPL6 = IAMP*EXP(ETA(11))**

**IF(FREQ.GE.7) AMPL7 = IAMP*EXP(ETA(12))**

**IF(FREQ.GE.8) AMPL8 = IAMP*EXP(ETA(13))**

**IF(FREQ.GE.9) AMPL9 = IAMP*EXP(ETA(14))**

**IF(FREQ.GE.10) AMPL10 = IAMP*EXP(ETA(15))**

**IF(FREQ.GE.11) AMPL11 = IAMP*EXP(ETA(16))**

**IF(FREQ.GE.12) AMPL12 = IAMP*EXP(ETA(17))**

**IF(FREQ.GE.13) AMPL13 = IAMP*EXP(ETA(18))**

**IF(FREQ.GE.14) AMPL14 = IAMP*EXP(ETA(19))**

**IF(FREQ.GE.15) AMPL15 = IAMP*EXP(ETA(20))**

**IF(FREQ.GE.16) AMPL16 = IAMP*EXP(ETA(21))**

**IF(FREQ.GE.17) AMPL17 = IAMP*EXP(ETA(22))**

**IF(FREQ.GE.18) AMPL18 = IAMP*EXP(ETA(23))**

**IF(FREQ.GE.19) AMPL19 = IAMP*EXP(ETA(24))**

**IF(FREQ.GE.20) AMPL20 = IAMP*EXP(ETA(25))**

**S1=1**

**$DES**

**E= (Emax*(A(2)**gamma))/(ec50**gamma+A(2)**gamma)**

**RIN1 = 0+EXP(LOG(AMPL1)-0.5*((T-P1)/SW)**2)*(1-E)**

**RIN2 = 0+EXP(LOG(AMPL2)-0.5*((T-P2)/SW)**2)*(1-E)**

**RIN3 = 0+EXP(LOG(AMPL3)-0.5*((T-P3)/SW)**2)*(1-E)**

**RIN4 = 0+EXP(LOG(AMPL4)-0.5*((T-P4)/SW)**2)*(1-E)**

**RIN5 = 0+EXP(LOG(AMPL5)-0.5*((T-P5)/SW)**2)*(1-E)**

**RIN6 = 0+EXP(LOG(AMPL6)-0.5*((T-P6)/SW)**2)*(1-E)**

**RIN7 = 0+EXP(LOG(AMPL7)-0.5*((T-P7)/SW)**2)*(1-E)**

**RIN8 = 0+EXP(LOG(AMPL8)-0.5*((T-P8)/SW)**2)*(1-E)**

**RIN9 = 0+EXP(LOG(AMPL9)-0.5*((T-P9)/SW)**2)*(1-E)**

**RIN10 = 0+EXP(LOG(AMPL10)-0.5*((T-P10)/SW)**2)*(1-E)**

**RIN11 = 0+EXP(LOG(AMPL11)-0.5*((T-P11)/SW)**2)*(1-E)**

**RIN12 = 0+EXP(LOG(AMPL12)-0.5*((T-P12)/SW)**2)*(1-E)**

**RIN13 = 0+EXP(LOG(AMPL13)-0.5*((T-P13)/SW)**2)*(1-E)**

**RIN14 = 0+EXP(LOG(AMPL14)-0.5*((T-P14)/SW)**2)*(1-E)**

**RIN15 = 0+EXP(LOG(AMPL15)-0.5*((T-P15)/SW)**2)*(1-E)**

**RIN16 = 0+EXP(LOG(AMPL16)-0.5*((T-P16)/SW)**2)*(1-E)**

**RIN17 = 0+EXP(LOG(AMPL17)-0.5*((T-P17)/SW)**2)*(1-E)**

**RIN18 = 0+EXP(LOG(AMPL18)-0.5*((T-P18)/SW)**2)*(1-E)**

**RIN19 = 0+EXP(LOG(AMPL19)-0.5*((T-P19)/SW)**2)*(1-E)**

**RIN20 = 0+EXP(LOG(AMPL20)-0.5*((T-P20)/SW)**2)*(1-E)**

**SECRETION = RIN1+RIN2+RIN3+RIN4+RIN5+RIN6+RIN7+RIN8+RIN9+RIN10+RIN11+RIN12+RIN13+RIN14+RIN15+RIN16+RIN17+RIN18+RIN19+RIN20**

**DADT(1)= KIN + SECRETION - KOUT*A(1)**

**CGH = A(1)**

**DADT(2) = -Kel*A(2)**

**$ERROR**

**IPRE = 0.00001**

**IF (F.GT.0) IPRE=F**

**Y=IPRE*(1+EPS(1))**

**$THETA**

**2.78 FIX ; kout ( /h)**

**0.185 FIX ; Baseline (mU/L)**

**7.86 FIX ; Individual amplitude (mU/L)**

**1.05 FIX ; A_0(1) (mU/L)**

**0.182 FIX ; SecretionSD (h)**

**3.4 FIX ; Exponent covariate relationship Amplitude**

**4.29 FIX ; Exponent covariate relationship Baseline**

**2.32 FIX ; Exponent covariate relationship SecretionSD**

**(0.01, 0.6,1) ; Emax**

**(0.01, 2 ) ; EC50**

**(0.01, 1 ) ; Gamma**

**$OMEGA**

**0.0699 FIX ; Kout**

**$OMEGA BLOCK(2)**

**0.406 ; Baseline**

**0.233 0.22 FIX ; AMP**

**$OMEGA**

**3.34 FIX ; A_0**

**0.0715 FIX; Secretion Width**

**$OMEGA BLOCK(1) 2.32 FIX; BOV on Amplitude 1-20**

**$OMEGA BLOCK(1) SAME (19)**

**$OMEGA**

**0 FIX ; EMAX**

**0.1 ; EC50**

**0 FIX ; GAMMA**

**$SIGMA**

**0.1 ; proportional error model**

**$EST PRINT=5 MAX=9999 METHOD=1 NSIG=3 SIGL=6 INTERACTION POSTHOC NOABORT MSFO=mfi**

**$COV PRINT=E**

**$TABLE ID TIME MDV AMT CMT CGH CWRESI IPRE PRED RES WRES NOAPPEND NOPRINT ONEHEADER FILE=EstimationResults.table**

***Goodness of fit plots – re-estimated model***


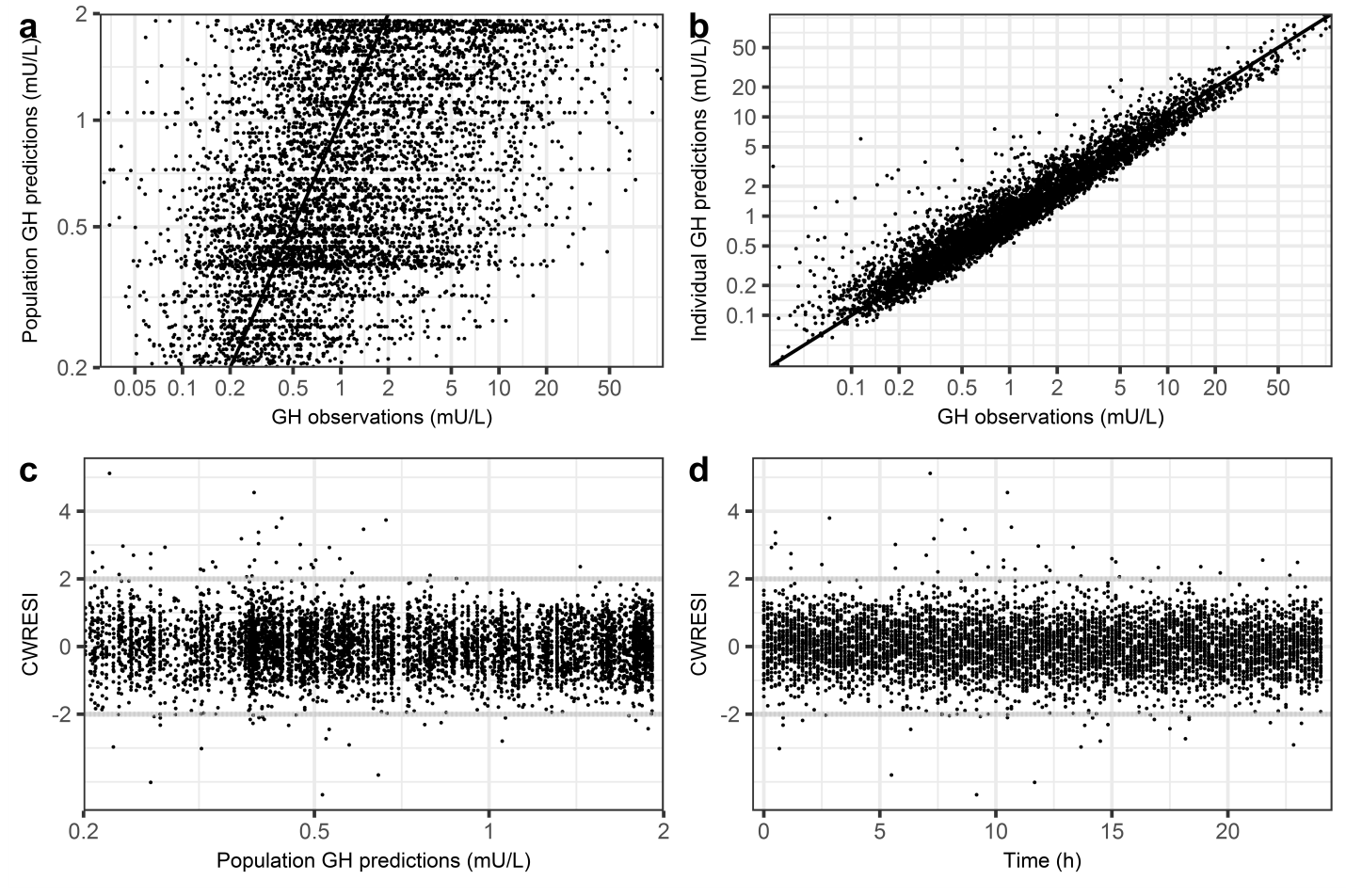


**a) Population GH model predictions versus simulated GH observations b) Individual GH model predictions versus simulated GH observations c) CWRESI versus population predictions d) CWRESI versus time (after dose). Black diagonal line indicates line of unity. Grey dashed horizontal lines indicate the [-2,2] interval.**
